# The complete mitochondrial genome of a yeti crab *Kiwa tyleri* Thatje, 2015 (Crustacea: Decapod: Anomura: Kiwaidae) from deep-sea hydrothermal vent

**DOI:** 10.1080/23802359.2017.1289347

**Published:** 2017-03-10

**Authors:** Dongsheng Zhang, Yadong Zhou, Hong Cheng, Chunsheng Wang

**Affiliations:** a Laboratory of Marine Ecosystem and Biogeochemistry, Second Institute of Oceanography, State Oceanic Administration, Hangzhou, China;; b State Key Laboratory of Satellite Ocean Environment Dynamics, Second Institute of Oceanography, State Oceanic Administration, Hangzhou, China

**Keywords:** *Kiwa tyleri*, complete mitogenome, Anomura, hydrothermal vent

## Abstract

The complete mitochondrial genome of *Kiwa tyleri* (Anomura, Chirostyloidea, Kiwaidae) was recovered by next generation sequencing. The mitogenome is 16,865 bp in length and contains 13 protein-coding genes (PCGs), 22 transfer RNAs (tRNAs), two ribosomal RNAs (rRNAs) and a 1525bp non-coding AT-rich region. This is the first mitogenome for the family Kiwaidae and the superfamily Chirostyloidea. The inversion of three consecutive genes (16S rRNA, tRNA-Val, 12S rRNA) was first reported for the Anomura. The phylogenetic tree indicated that Kiwaidae was close to Paguroidea and Lithodoidea rather than Galatheoidea.

Kiwaidae is a small family of anomuran species, which has been highly specialized for the extreme environment of chemosynthetic ecosystems in East Pacific, Southern Ocean and Southwest Indian Ridge (Roterman et al. 2013). Its genetic information has great potential for studies on biogeographic history of vent-endemic fauna (Thatje et al. 2015; Roterman et al. 2016). In the present study, we determined the mitogenome of *Kiwa tyleri*, to provide the first mitogenomes in Kiwaidae.

Specimens were collected from Longqi vent field on SWIR by the manned submersible Jiaolong and deposited in the Laboratory of Marine Ecosystem and Biogeochemistry, Second Institute of Oceanography, Hangzhou. Genomic DNA was extracted using a QIAamp DNA Mini Kit (Qiagen, CA, USA). The pair-end (2X450bp) sequencing library was prepared and launched on Illumina Hiseq4000 sequencer (Illumina, CA, USA). About 8 Gb raw data were *de novo* assembled by CLC Genomics Workbench v8.0 (Qiagen, CA, USA) and ABySS 1.5.2 (Simpson et al. 2009). The complete mitogenome obtained by SeqMan package (LaserGene 8.1.3, DNASTAR, Inc. WI, USA) from assembly result was annotated with MITOS (Bernt et al. 2013) and the borders of protein-coding genes (PCGs) were adjusted manually by comparison with sequenced Anomura mitogenomes. Alignments of 13 PCGs were performed using the program MAFFT (Katoh & Standley 2013). Phylogenetic analysis was conducted by RAxML (Stamatakis 2014) using concatenated nucleotide sequences of 13 PCGs.

The complete mitochondrial genome of *K*. *tyleri* is 16,865 bp in length (Genbank accession number: KY423514), and contains 13 PCGs, 22 transfer RNAs (tRNAs), 2 ribosomal RNAs (rRNAs) and a non-coding AT-rich region of 1525bp in length. The base composition of the mitogenome was 37.9% for A, 41.4% for T, 12.6% for C and 8.1% for G. COX1, ND3, ATP8, ND5, ND4L, ND6 contain ATT as the start codon; COX2, ATP6, COX3 and CYTB contain ATG as the start codon; ND2 and ND1 contain ATA as the start codon; ND4 contains GTG as the start codon. All PCGs contain TAA as the stop codon except for incomplete stop codons of TA or T of ND4 and ND5.

PCGs order of *K*. *tyleri* is identical to *Paralithodes brevipes*, *P. camtschaticus*, *Lithodes nintokuae* and *Pagurus longicarpus*. However, *K*. *tyleri* contains an inversion of three consecutive genes (16S rRNA, tRNA-Val and 12S rRNA) compared to all the other sequenced mitogenomes of the Anomura. Maximum likelihood tree (Figure 1) showed that *K*. *tyleri* was grouped with the Paguroidea and Lithodoidea as reported (Bracken-Grissom et al. 2013).

**Figure 1.**
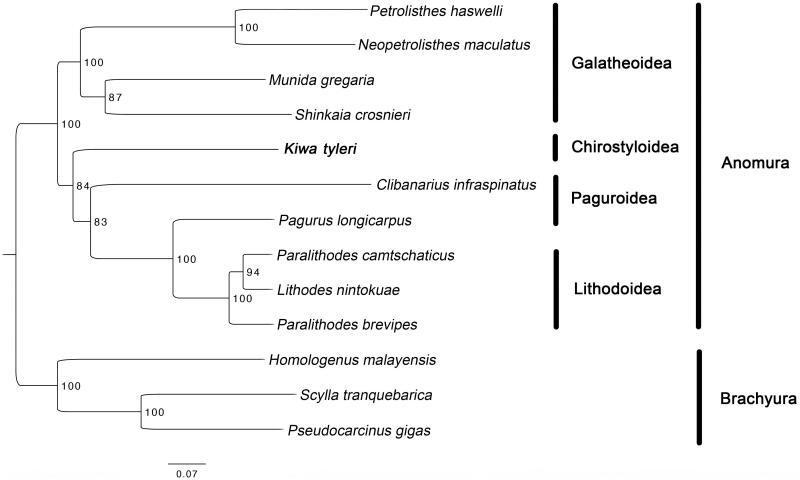
Maximum likelihood (ML) tree based on Kiwa tyleri with other 9 species from Anomura and 3 species from Brachyura. Bootstrap support values were generated with a rapid bootstrapping algorithm for 1000 replicates. The following mitogenomes were used in this analysis: *Petrolisthes haswelli* (LN624374), *Neopetrolisthes maculatus* (NC_020024), *Munida gregaria* (KU521508), *Shinkaia crosnieri* (EU420129), *Clibanarius infraspinatus* (LN626968), *Pagurus longicarpus* (AF150756), *Paralithodes camtschaticus* (JX944381), *Lithodes nintokuae* (AB769476), *Paralithodes brevipes* (AB735677), *Homologenus malayensis* (NC_026080), *Scylla tranquebarica* (NC_012567) and *Pseudocarcinus gigas* (NC_006891).
